# Exploring Publicly Accessible Optical Coherence Tomography Datasets: A Comprehensive Overview

**DOI:** 10.3390/diagnostics14151668

**Published:** 2024-08-01

**Authors:** Anastasiia Rozhyna, Gábor Márk Somfai, Manfredo Atzori, Delia Cabrera DeBuc, Amr Saad, Jay Zoellin, Henning Müller

**Affiliations:** 1 Informatics Institute, University of Applied Sciences Western Switzerland (HES-SO), 3960 Sierre, Switzerland; 2 Medical Informatics, University of Geneva, 1205 Geneva, Switzerland; 3 Department of Ophthalmology, Stadtspital Zürich, 8063 Zurich, Switzerland; 4 Spross Research Institute, 8063 Zurich, Switzerland; 5 Department of Neuroscience, University of Padua, 35121 Padova, Italy; 6 Bascom Palmer Eye Institute, Miller School of Medicine, University of Miami, Miami, FL 33136, USA; 7 The Sense Research and Innovation Center, 1007 Lausanne, Switzerland

**Keywords:** OCT, optical coherence tomography, datasets, data, open data, data sharing, data analysis

## Abstract

Artificial intelligence has transformed medical diagnostic capabilities, particularly through medical image analysis. AI algorithms perform well in detecting abnormalities with a strong performance, enabling computer-aided diagnosis by analyzing the extensive amounts of patient data. The data serve as a foundation upon which algorithms learn and make predictions. Thus, the importance of data cannot be underestimated, and clinically corresponding datasets are required. Many researchers face a lack of medical data due to limited access, privacy concerns, or the absence of available annotations. One of the most widely used diagnostic tools in ophthalmology is Optical Coherence Tomography (OCT). Addressing the data availability issue is crucial for enhancing AI applications in the field of OCT diagnostics. This review aims to provide a comprehensive analysis of all publicly accessible retinal OCT datasets. Our main objective is to compile a list of OCT datasets and their properties, which can serve as an accessible reference, facilitating data curation for medical image analysis tasks. For this review, we searched through the Zenodo repository, Mendeley Data repository, MEDLINE database, and Google Dataset search engine. We systematically evaluated all the identified datasets and found 23 open-access datasets containing OCT images, which significantly vary in terms of size, scope, and ground-truth labels. Our findings indicate the need for improvement in data-sharing practices and standardized documentation. Enhancing the availability and quality of OCT datasets will support the development of AI algorithms and ultimately improve diagnostic capabilities in ophthalmology. By providing a comprehensive list of accessible OCT datasets, this review aims to facilitate better utilization and development of AI in medical image analysis.

## 1. Introduction

Artificial intelligence (AI) has become one of the most innovative fields that have revolutionized healthcare [1]. Recent progress in deep learning (DL) enables the identification, classification, and quantification of patterns within medical images. DL algorithms can detect biomarkers and abnormalities in medical images, aiding in early disease detection and computer-aided diagnosis [2]. These algorithms heavily rely on diverse amounts of labeled data for training robust models. The performance of DL models is directly influenced by the quality, quantity, and diversity of the data used for training [3]. Access to biomedical datasets is crucial for ensuring the generalizability and effectiveness of DL models. Annotated, labeled datasets facilitate the training process by providing ground truth labels of specific medical conditions. Thus, the availability and quality of data are vital to reduce the problem of overfitting and to improve the generalization performance of DL models [4]. Privacy, ethical concerns, and restricted access are significant limitations of data sharing in the medical imaging field [5]. The absence of standardized protocols for data sharing and the use of different formats and standards make it challenging to integrate and share data across healthcare and research institutions.

In ophthalmology, there are various imaging modalities for the detection, diagnosis, and management of eye conditions. These modalities include Color Fundus Photography (CFP), Fundus fluorescein angiography (FFA), Indocyanine green angiography (ICGA), Optical coherence tomography (OCT), Optical coherence tomography angiography (OCTA), and Confocal scanning laser ophthalmoscopy (CSLO) [6]. Optical coherence tomography (OCT) is widely employed for early diagnosis and guiding therapeutic decisions in various retinal diseases and shows promise in detecting subtle retinal vascular changes [7]. OCT is an important imaging modality that allows for the high-resolution, non-invasive observation of retinal structures with an almost cellular resolution. OCT works by emitting light waves into the eye and measuring the delay and intensity of the reflected light. When light reflects off different layers of the retina, it creates an interference pattern that can be detected and analyzed. OCT stands out as the most widely used diagnostic tool for retinal disorders because it is a non-invasive, distinctive, and high-resolution assessment of tissues. Its ability to directly correspond to the histological features of the retina allows for achieving an axial resolution of 2–3 µm within tissues [8].

The vast amount of ocular imaging data produced in clinical and research environments presents a significant challenge for clinicians and researchers in effectively sharing images, even with patients. Despite advancements in imaging technology, this struggle persists. Given the reliance on big data in modern research, widespread adoption of standardized practices would substantially improve digital workflow efficiency. Various studies have highlighted the need for uniform, cohesive, standardized reporting guidelines [9].

The Advised Protocol for OCT Study Terminology and Elements (APOSTEL) recommendations were introduced in 2016 and updated in 2021 [10]. These guidelines offer a concise 9-point checklist outlining essential elements to include when reporting quantitative retinal OCT studies. Other studies have introduced recommendations for OCT/OCTA nomenclature and reporting in retinal vascular diseases [11,12]. Standardization and interoperability are key for advancing research and introducing breakthroughs in the field [13,14].

The National Eye Institute’s (NEI) recent notice (NOT-EY-24-006) emphasizes the importance of using common file formats and metadata standards for ocular imaging in clinical and nonclinical research [15,16]. This initiative aims to facilitate data standardization, interoperability, and the sharing of raw data and metadata, thereby addressing some of the key challenges identified in our study. The NEI strongly encourages the adoption of standards like the Digital Imaging and Communications in Medicine (DICOM) format, which will greatly enhance the digital workflow, allow for the sharing of large datasets, and create substantial training sets for AI-based research. These standardized practices are essential for advancing the field and ensuring that research findings are reproducible and reliable. By adhering to these standardized data-sharing practices, the research community can significantly improve the utility and impact of OCT datasets. This will not only enhance the development of AI tools but also contribute to more effective and efficient patient care in ophthalmology. Therefore, future research and data-sharing efforts must align with the guidelines and recommendations set forth by the NEI, promoting a more collaborative and standardized approach to ocular imaging research.

Regarding medical image open-access datasets, Johann Li et al. provided a collection of medical image datasets and the DL challenges associated with these data. In particular, the article discusses the significance of ophthalmic datasets, emphasizing the critical role of eye health in preventing blindness. It provides an overview of various datasets with a focus on modalities such as CFP and OCT, as well as surgical videos. The datasets are categorized based on analysis tasks. In the paper, it is noted that only five datasets were specifically focused on OCT. Three of the mentioned datasets were linked to specific DL challenges and were featured as part of conferences, while the remaining two were publicly accessible. However, the datasets associated with challenges require additional data agreements and restrict their use [17].

Khan et al. found 94 open-access ophthalmological imaging datasets. Among those datasets, OCT datasets comprised 15 datasets out of 94. Out of the 15 OCT datasets mentioned, only 11 were accessible, while the remaining 4 were not available, despite the corresponding links provided in the review paper. These four links were either inaccessible or resulted in errors during use (during review in May 2024) [18]. Despite the availability of numerous datasets, challenges persist in accessing and utilizing them effectively, as evidenced by the limited accessibility of OCT datasets. Moving forward, addressing these challenges and promoting open access to diverse datasets will be essential for advancing research and improving patient outcomes.

For the above reasons, in our work, we aim to address the growing need for well-annotated OCT datasets for diagnosing eye diseases. Our goal is to create an updated list of existing OCT image datasets for researchers to gain easier access to data for training algorithms, addressing the accessibility issues highlighted in previous works by providing direct links and verification of dataset availability. Also, we aimed to identify gaps in available datasets to address these issues and provide updated resources for researchers. Assessing the completeness and quality of metadata associated with each dataset is crucial for the effective utilization and integration of these datasets in AI research.

## 2. Materials and Methods

In this section, the methodology employed during the review is discussed in detail. The processes and techniques utilized to gather, analyze, and interpret the data are outlined, providing transparency and insight into the research approach.

### 2.1. Search Strategy and Selection Criteria

The search strategy and selection criteria for the dataset review were designed to ensure comprehensive coverage of ophthalmological imaging datasets while maintaining methodological precision. The search consisted of several parts. The review methodology aimed to examine two primary data sources: publicly accessible datasets and research papers containing references to accessible datasets within their findings. We initially employed established tools to explore repositories containing OCT images.

**Figure 1:**
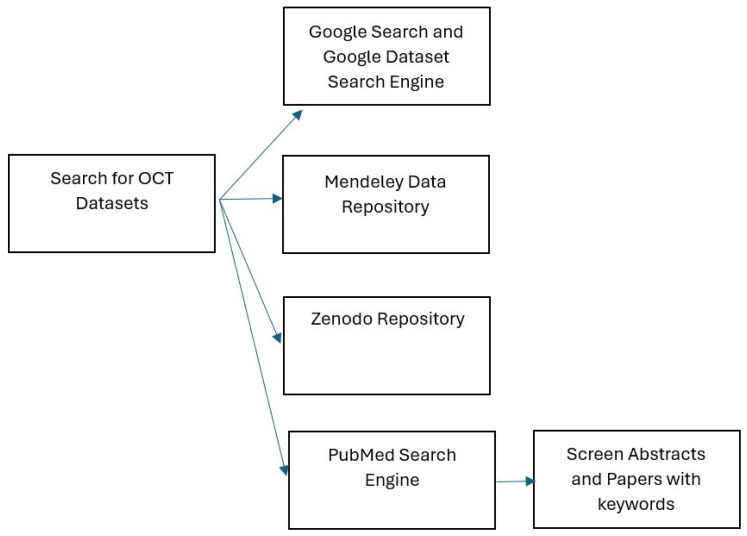
The dataset search with detailed search engines.

Figure 1 demonstrates the dataset search with detailing search engines. This involved targeted searches on platforms such as the Google Dataset search engine and the standard Google search engine, using varied keywords centered on terms like “retina” and “OCT”, along with keywords like “dataset”, “database”, and “repositories”. Additionally, we utilized the Mendeley Data repository, a platform enabling researchers to organize, share, and manage their data systematically. Another approach involved searching the Zenodo repository using the same keywords. Zenodo is an open repository established as part of the European OpenAIRE program and operated by CERN. Finally, we conducted searches on PubMed, a search engine primarily accessing the MEDLINE database, a comprehensive biomedical database maintained by the National Library of Medicine (NLM) in the United States. PubMed often contains links to datasets utilized in published studies.

The review process entailed screening titles and abstracts for articles detailing OCT image datasets and studies employing OCT for retinal diagnosis, particularly those utilizing datasets to train machine learning algorithms. We also referred to the insights provided in the evaluation of publicly accessible ophthalmological imaging datasets [18] to validate the mentioned datasets and their current status and availability.

For the datasets to be eligible for data extraction, they needed to contain retinal OCT scan images. If datasets had no retinal OCT, they were excluded. Datasets with text or numeric-only data were excluded. Datasets currently inaccessible but previously described as open-access were excluded. The search was conducted from March to May 2024.

### 2.2. Dataset Access

The accessibility of the data varied based on the levels of access to the datasets, ranging from complete accessibility to being available upon request after contacting the authors.

We defined the access levels as follows:

- Open access. No requirements for access. No preconditions for accessing or using data in a certain way, allowing users to retrieve the data freely;
- Open access with restrictions. Requirements for access: completing the form, account registration, sending an email to authors and getting approval from them, and obtaining the assessment or special code for decryption of the images. The restriction on application use for instance datasets associated with conference challenges;
- Restricted access. Additional legal agreements, meeting special requirements, payments for access, etc.;
- Not accessible. The datasets categorized as not accessible are those for which the data were inaccessible or unavailable for use due to privacy or ethical concerns. This category includes the datasets that were initially described as accessible but has since become inaccessible due to various reasons, such as inactive links or lack of response from authors, thereby blocking access to the data.

Throughout the search process, our primary focus was on investigating the accessibility and usability of these datasets, with particular emphasis on those that are openly accessible or available with restrictions. We investigated the completeness of metadata and any additional information associated with those datasets. After gaining access, we examined each dataset by downloading them to extract details regarding file status, sizes, and any additional artifacts present in the images. The majority of the accessible datasets were provided as compressed files.

## 3. Results

**Figure 2:**
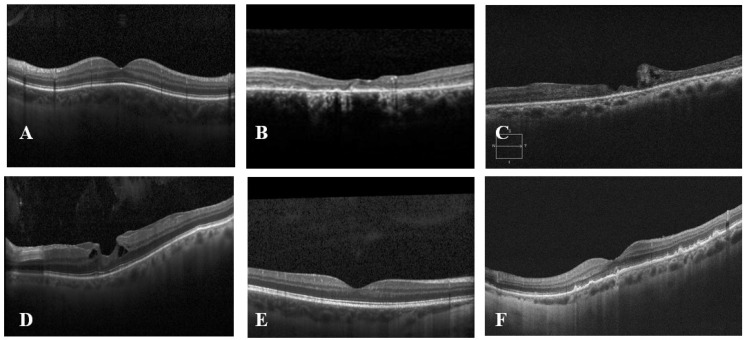
Exemplary sample images from datasets. (**A**)—Kermany et al.; (**B**)—Data of rare retinal disease; (**C**)—OCTID; (**D**)—Labeled Retinal Optical Coherence Tomography Dataset for Classification; (**E**)—OCT MS and HC data; (**F**)—Duke OCT.

In this study, a total of 23 publicly accessible OCT imaging datasets were identified. Out of the 23 datasets analyzed, 6 were discovered on Mendeley Data repository, 8 were sourced through Google Search engines, 7 were obtained from PubMed, and 2 were found on Zenodo repository. Each dataset was analyzed based on several key factors, including the country of origin, the number of images, annotations, image resolution, and image format. Figure 2 demonstrates random sample selection from the found datasets.

### 3.1. Quality of Images

A visual assessment of the image quality across datasets is crucial to understanding diagnostic and research findings. There are factors that contribute to the quality of OCT images, including image resolution, signal strength, noise level, artifacts, and overall image clarity. The higher-resolution images give better visualization of anatomical structures and abnormalities. OCT images are often influenced by several artifacts, and speckle noise is one of the most common artifacts in OCT imaging. Speckle noise appears as grainy or granular patterns on the image and can obscure important details, affecting the interpretation and analysis of the image [19]. Thus, many papers evaluate the quality of the images, and this evaluation is performed by human grading.

From Figure 2, it can be observed that some datasets have tilted images. This can be a result of incorrect, improper alignment in the imaging system. This misalignment can occur due to various reasons, such as improper positioning of the patient’s head during image acquisition or inaccuracies in the OCT device itself [20].

Table 1 summarizes publicly accessible OCT imaging datasets, including information on their origin country, accessibility status, image number, representation of diseases, file format, and resolution.

Table A1 from the Appendix A has additional information for dataset access (e.g., links for access).

### 3.2. Metadata

The completeness and availability of the metadata related to the found datasets is crucial in their usability. Metadata provides essential information about other data, making them easier to find, use, and manage. Metadata standards ensure that datasets can be integrated and compared across different studies and institutions. This is particularly important in ophthalmology, where combining data from multiple sources can enhance the robustness of research findings and support large-scale studies [44].

#### 3.2.1. Disease Representation

**Figure 3:**
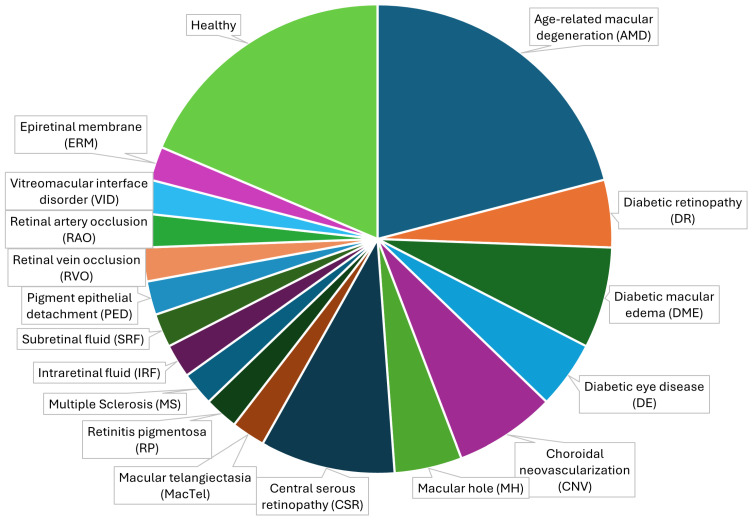
Disease representation across datasets.

Figure 3 demonstrates the disease representation across the available datasets. Conditions like diabetic eye disease (DE) and AMD are over-represented in the datasets but with fewer occurrences than healthy eyes. This suggests a limited number of diseases that are present in the publicly available datasets. The datasets provide a representation of glaucoma as well. The distribution of diseases represented in the dataset is influenced by several factors, including the prevalence of these conditions in the general population, as well as the focus of clinical studies. AMD and diabetic retinopathy (DR) are among the most prevalent causes of vision loss in older adults, which likely accounts for their significant representation in the datasets. Wong et al. report that AMD affects millions globally [45].

The high representation of conditions such as AMD and DR can also be attributed to the focus of many ophthalmological studies on these prevalent diseases. Researchers often prioritize these conditions due to their substantial public health burden and the potential for significant clinical improvements with better management and treatment options [46]. Biases in diagnosis and reporting can also play a role. Conditions that are more easily diagnosed or have well-established diagnostic criteria, such as AMD, DR, and DME, might be reported more frequently in contrast to rarer conditions or those with less clear diagnostic criteria.

#### 3.2.2. Origin of Datasets

Geographical bias in datasets is a significant issue that can impact the generalizability and applicability of research findings. The over-representation of certain regions can limit the generalizability of research findings, as the results may not apply to populations in the underrepresented areas [47].

**Figure 4:**
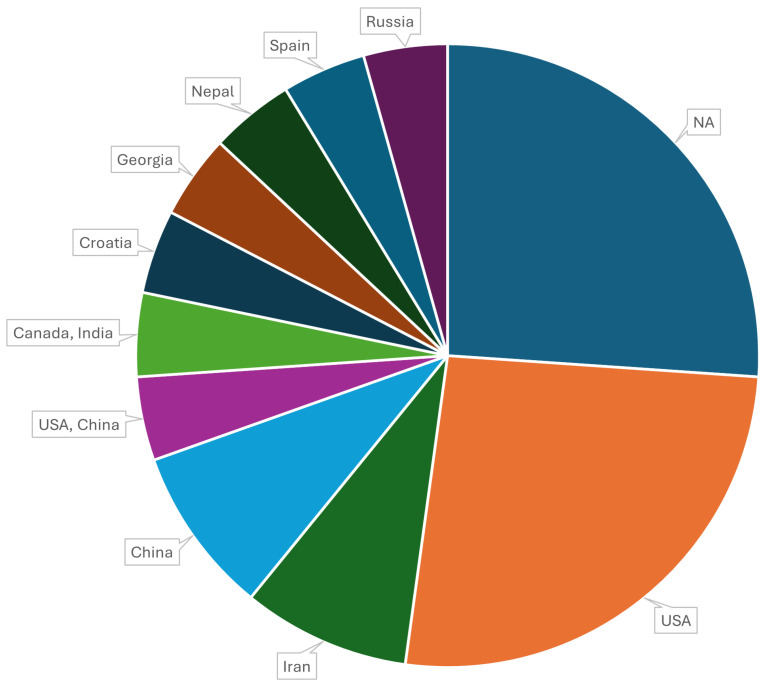
Origin of datasets.

Figure 4 displays the distribution of OCT datasets by country of origin. During our study, no datasets were excluded based on country of origin. From the analysis, it is evident that the United States has contributed the highest number of datasets, followed by China. Several datasets have origins that include multiple countries with collaborative research. Additionally, there are datasets with unspecified origins labeled as “NA”. In metadata, it is either a lack of available information or not mentioning the country of acquisition.

Adopting open data policies can improve access to datasets from diverse geographical regions. Open data initiatives can facilitate the sharing and integration of data.

In our study, we noticed notable geographical bias in the absence of datasets from large parts of the world, including Africa, South America, and Oceania, indicating a significant geographical bias. Differences in healthcare access and the presence of screening programs can influence dataset composition.

#### 3.2.3. Acquisition Protocols

Table 2 summarizes the additional metadata such as the number of patients and the used imaging device. The Heidelberg Spectralis SD-OCT imaging system (Heidelberg Engineering) is the most commonly used acquisition scanner, appearing in 10 out of 20 datasets. This is approximately 50% of the datasets. Cirrus HD-OCT machine (Carl Zeiss Meditec) appears in 3 out of 20 datasets, constituting approximately 15% of the datasets. SD-OCT imaging system (Bioptigen) and OCT Cirrus 500 (Carl Zeiss Meditec) each appear in 1 out of 20 datasets, contributing approximately 5% of the datasets individually.

There are also instances where the acquisition scanner information is not available (NA), which indicates that the scanner used was either not specified or was not applicable for synthetic images or datasets where acquisition details were not provided. The Heidelberg Engineering Spectralis was the most used choice for acquiring OCT images.

#### 3.2.4. Laterality

Laterality refers to the distinction between images acquired from the left eye versus the right eye. This distinction is important for properly interpreting and comparing OCT data, especially in ophthalmology where structural differences between the eyes may exist due to anatomical variations or pathological conditions. There are asymmetries between the left- and right-eye OCT images [48].

Laterality is often indicated using abbreviations such as OD (oculus dexter) for the right eye and OS (oculus sinister) for the left eye. Occasionally, the term OU (oculi uterque) indicates both eyes.

Out of the 22 OCT datasets analyzed, information about laterality is provided for only 6 datasets, representing approximately 27% of the total datasets. This limited availability of laterality information underscores the importance of comprehensive documentation in OCT studies, particularly regarding the distinction between left- and right-eye images for accurate interpretation and clinical relevance.

#### 3.2.5. Image Format and Image Resolution

Among the commonly used file formats, JPEG, TIFF, and PNG were the most prevalent options. The choice of file format could potentially impact the quality and effectiveness of the images captured. Furthermore, certain datasets provide images in formats such as MAT and NumPy, which may necessitate specialized software or libraries for handling and processing. The discrepancy in image resolution across the datasets is notable, with sizes varying from 64 × 128 to 512 × 1024 pixels. Image resolution was a crucial aspect assessed to determine the quality and clarity of the images, with higher resolutions generally allowing for a more detailed analysis. The image format was examined to ensure compatibility with common image processing and analysis tools, facilitating seamless integration into research workflows. To assess the quality of images across the datasets, we decided to take three of the most similar datasets by disease and acquisition setup and compare the pixel intensities across the healthy subsets from the datasets. By analyzing the histograms of pixel intensities and by examining the shape of these histograms, we can identify key characteristics such as uniformity, dynamic range, and the presence of noise or artifacts. High-quality images exhibit a wide distribution of pixel intensities, indicating good contrast and the effective use of the available dynamic range. Comparing these histograms across different datasets helps to check consistency and detect possible biases in the dataset. Thus, we decided to take the Kermany dataset, OCTID dataset, and Labeled Retinal OCT Dataset for Classification. All of them were compared by healthy class. In OCT images, pixel-intensity histograms reveal information about the structure and composition of tissues within the scanned area. The variability in pixel intensity distribution histograms demonstrates that the images may show diverse characteristics, influenced by factors such as lighting conditions, acquisition settings, or differences between the datasets.

**Figure 5:**
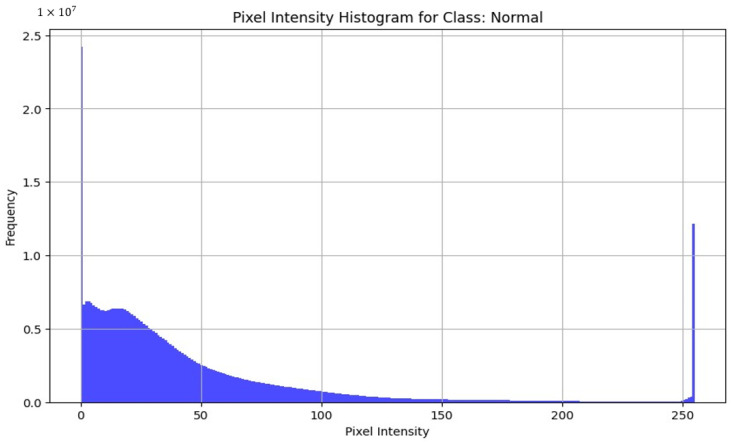
Histogram for Kermany dataset.

**Figure 6:**
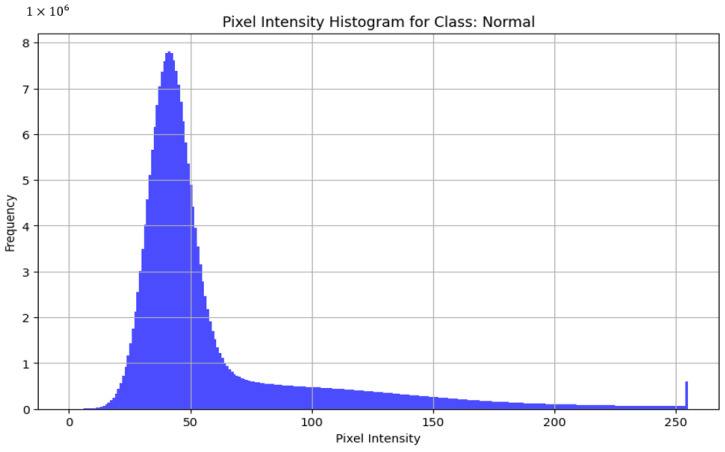
Histogram for OCTID dataset.

**Figure 7:**
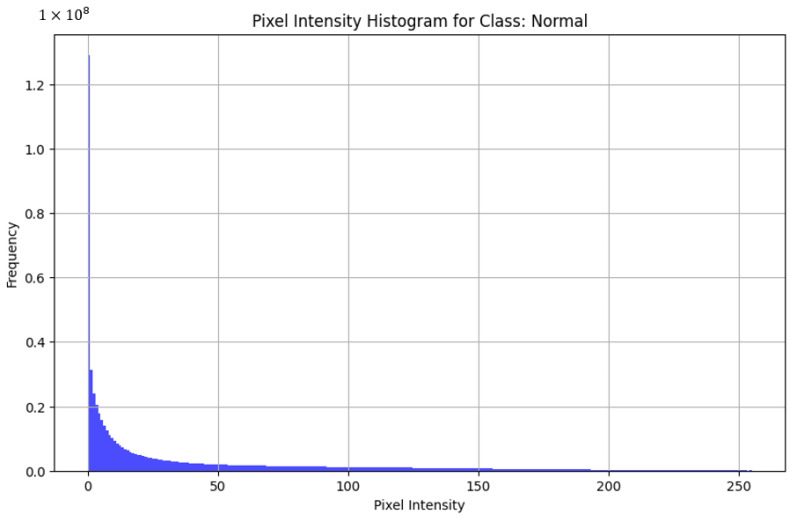
Histogram for Labeled Retinal OCT Dataset for Classification dataset.

Figure 5 reveals a high frequency of low-pixel intensity values, indicating a significant number of dark pixels, which is characteristic of OCT images where the background is dark. There is a noticeable peak at the higher end of the pixel intensity spectrum, suggesting bright reflections from retinal layers. The gradual decrease in frequency as pixel intensity increases from 0 suggests a smooth transition from darker to brighter regions, indicating a uniform distribution and good image quality. This exhibits a wide dynamic range with minimal noise, making it suitable for model training. Figure 6 with the histogram of the OCTID dataset exhibits a peak around mid-range pixel intensities, indicating a balanced contrast with a distribution of light and dark regions. There is a sharp decline in frequency towards higher pixel intensities, with a small peak at the very end, suggesting bright spots that might be reflections or artifacts. The overall shape of the histogram suggests high-quality images with a good contrast. Figure 7 presents the histogram showing a very high frequency of low-pixel intensity values, indicating many dark regions. This distribution can demonstrate a potential issue with image uniformity, possibly due to noise. While this dataset has a good dynamic range, the variability in pixel intensity suggests the need for careful preprocessing.

### 3.3. Application of OCT Datasets

DL techniques are widely applied to OCT images. The most common tasks include disease classification, detection of abnormalities, and segmentation of retinal layers.

#### 3.3.1. Classification

Classification tasks using OCT datasets involve categorizing images into distinct diseases or condition classes. For instance, the Kermany et al. dataset [21] has been utilized for numerous purposes, including classification [49,50]. The retinal OCT images of rare diseases [22] were used in [51] for the improvement of detecting rare retinal diseases using OCT images by DL with a GAN technique. OCTID [23] in [52,53] was used to train multi-modal image classification and improvement of retinal pathology classification. The network for noiseless image algorithm is proposed in [54] for sorting the clear images from Corneal-OCT Dataset [24]. A Composite Retinal Fundus and OCT Dataset [25] was performed for the screening of macular and glaucomatous disorders. Labeled Retinal OCT Dataset for Classification [27] was used in [55], introducing a multi-scale CNN based on the feature pyramid network structure for automated classification of retinal pathologies. Duke OCT [28] was used in the classification algorithm for defining quantitative indicators for the presence of intermediate AMD.

#### 3.3.2. Detection

Detection tasks focus on identifying specific abnormalities or features within OCT images. The Retina OCT Glaucoma dataset [30] was applied to the detection of glaucoma with a feature-agnostic approach [56]. The OLIVES Dataset [31] was used in [57] for the simultaneous automatic recognition of ophthalmic biomarkers. In 2014, Srinivasan [33] was used to fully automate DME and dry AMD detection. THOCT1800 [40] was used for the automatic detection of retinal regions. OCHID: An OCT Choroid Segmentation Dataset [41] was used in several works that focus on detection in retinal images using multi-scale deep-feature sparse coding [58].

#### 3.3.3. Segmentation

Segmentation tasks involve delineating different structures within OCT images, such as retinal layers. In 2012, Fang [34] was used in a sparsity-based denoising work. Duke 2015 [32] was used for kernel regression-based segmentation. Data on OCT and Fundus images [29] were used for the evaluation of an automated segmentation algorithm for the extraction of retinal layers [59]. The AROI database [26] is often used for a joint retinal layer and fluid segmentation. OCT MS and HC data [35] are often used in segmentation tasks [60]. OIMHS [36] was used in an image segmentation network that integrates spectrum information [61]. The Retouch dataset [37] was used in [62] to develop and validate deep learning models for segmenting retinal structures. The University of Auckland Dataset [38] and retinal image dataset [39] were utilized for retinal layer segmentation for patients with AMD.

The CLOUD Dataset [42] was used to automatically identify the cornea–contact lens relationship. OCTDL: Optical Coherence Tomography Dataset for Image-Based Deep Learning Methods [43] was used in works such as developing foundational models for ophthalmic images [63]. Each of these datasets and corresponding studies demonstrates the critical role of DL in enhancing the analysis and interpretation of OCT images for various medical applications.

## 4. Discussion

Our results highlight several critical aspects of OCT imaging datasets, underscoring the importance of greater diversity in disease representation among these datasets. The over-representation of certain diseases like DE and AMD in publicly accessible datasets suggests a potential bias towards these conditions. This bias may limit the development of AI models that can generalize well to less common eye diseases. Future efforts should focus on creating and sharing datasets that represent a broader range of conditions. Establishing standardized file formats and resolutions for OCT imaging datasets is crucial for facilitating a seamless data integration and analysis. Furthermore, the variability in image resolutions across datasets may impact the performance of algorithms trained on these data. Additionally, the incomplete metadata associated with many datasets highlights the need for standardized metadata documentation. This documentation should include acquisition protocols, laterality, image quality metrics, and detailed annotations to enhance dataset usability and reproducibility. Geographical bias is another critical issue identified in our study. The disproportionate datasets from certain regions, particularly the United States and China, may limit the applicability of research findings to global populations. To address this, international collaboration initiatives should be promoted to support the collection and sharing of OCT imaging data from underrepresented regions. The paper in [64] demonstrates the effectiveness of transfer learning in adapting models across diverse populations to predict refractive errors and corneal curvature from OCT images, addressing performance issues across different ethnic populations by adapting models pre-trained on a Korean dataset and validated on an Indian dataset. The adapted models showed a significantly improved accuracy compared to non-adapted models. This study demonstrates the potential of transfer learning to enhance the applicability of AI models in multi-ethnic contexts, highlighting the need for further research with larger and more diverse datasets. By leveraging data from different ethnic groups and various OCT devices, it is possible to develop more robust and generalizable models. This approach ensures that the developed AI models are capable of providing accurate diagnostics across a wide range of populations and imaging conditions.

Ethical and privacy concerns remain significant barriers to data sharing in medical imaging. The development of standardized protocols for data anonymization and secure data sharing is essential to address these concerns. Implementing federated learning approaches, where AI models are trained on data from multiple institutions without sharing the actual data, could also help.

In summary, while there are valuable OCT imaging datasets publicly accessible, a prevalent issue is that many publicly accessible databases remain obscure, primarily because they lack visibility, accessibility, transparency, and comprehensive data descriptions.

## 5. Conclusions

In conclusion, while OCT imaging datasets offer great potential for advancing AI-based research in ophthalmology, several challenges need to be addressed to maximize their impact. These challenges include the need for greater disease diversity, standardized file formats, comprehensive metadata documentation, and strategies to mitigate geographical bias. Addressing these issues will require collaborative efforts among researchers and clinicians to promote open access to diverse and well-documented OCT imaging datasets. Future research should focus on developing standardized protocols for data collection, annotation, and sharing to enhance the utility and interoperability of OCT datasets. This should, in the long term, lead to the development of clinical tools that can increase the quality of patient care, while reducing healthcare costs and personnel workload.

## Figures and Tables

**Table 1:** Publicly available OCT imaging datasets.

Dataset Name	Country	Access	Number of B-Scans	Eye Disease Details	File Details
Kermany et al. [21]	USA, China	Open access	109,312	DE, CNV, Drusen, Healthy	JPEG, 512 × 496
The retinal OCT images of rare diseases [22]	NA	Open access	119	CSR, MacTel, MH, Stargadt’s disease, RP	PNG, min. 290 × 277–max. 523 × 525
OCTID [23]	Canada, India	Open access with restrictions	572	Healthy, MH, AMD, CSR, DR	JPEG, 512 × 1024
Corneal-OCT Dataset [24]	Iran	Open access with restrictions	41	NA	MAT, 240 × 748
A Composite Retinal Fundus and OCT Dataset [25]	NA	Open access	768	DME, Acute CSR, Chronic CSR, Glaucoma, AMD	JPEG, 760 × 576
AROI database [26]	Croatia	Open access with restrictions	3200	Neovascular AMD	PNG, 1024 × 512
Labeled Retinal OCT Dataset for Classification [27]	Iran	Open access	16,822	CNV, Drusen, Healthy	JPEG, TIFF, 768 × 496
Duke OCT [28]	USA	Open access	35,400	AMD	MAT, 1001 × 1001
Data on OCT and Fundus Images [29]	NA	Open access	50	Healthy, Glaucoma	JPEG, 951 × 456
Retina OCT Glaucoma dataset [30]	NA	Open access	884	Healthy, Glaucoma	NumPy, 64 × 128
OLIVES Dataset [31]	Georgia	Open access	49	DR, AMD	PNG, TIFF, 504 × 496
Duke 2015 [32]	USA	Open access	110	DME	MAT, 512 × 740
2014 Srinivasan [33]	USA	Open access	3231	DE, AMD, Healthy	TIFF, 512 × 496
2012 Fang [34]	USA	Open access	51	AMD, Healthy	TIFF, 280 × 1000
OCT MS and HC data [35]	USA	Open access	1715	MS, Healthy	VOL, 496 × 1024
OIMHS [36]	China	Open access	3859	MH	JPEG, 1024 × 512
Retouch dataset [37]	NA	Open access with restrictions	11,334	IRF, SRF, PED	Raw Binary file, 512 × 1024, 512 × 496, 512 × 885 or 512 × 650
University of Auckland Dataset [38]	Nepal	Open access with restrictions	70,200	NA	VOL, 320 × 992, 1008 × 596
Retinal image dataset [39]	USA	Open access with restrictions	NA	AMD	VOL, 496 × 768 × 121
THOCT1800 [40]	China	Open access	1800	AMD, DME, Healthy	JPEG, 756 × 121
OCHID: An OCT Choroid Segmentation Dataset [41]	NA	Open access with restrictions	1920	Healthy	VOL, 992 × 512
CLOUD Dataset [42]	Spain	Open access with restrictions	112	NA	NA
OCTDL: Optical Coherence Tomography Dataset for Image-Based Deep Learning Methods [43]	Russia	Open access	2064	AMD, DME, RVO, RAO, VID, ERM	JPEG, 1100 × 410

**Table 2:** Acquisition metadata.

Dataset Name	Number of Patients	Device
Kermany et al. [21]	5319	Heidelberg Spectralis SD-OCT imaging system (Heidelberg Engineering)
The retinal OCT images of rare diseases [22]	NA (Synthetic images)	NA (Synthetic images)
OCTID [23]	NA	Cirrus HD-OCT machine (Carl Zeiss Meditec)
Corneal-OCT Dataset [24]	NA	Heidelberg Spectralis SD-OCT imaging system (Heidelberg Engineering)
A Composite Retinal Fundus and OCT Dataset [25]	64	NA
AROI database [26]	24	Zeiss Cirrus HD OCT 4000 device
Labeled Retinal OCT Dataset for Classification [27]	441	NA
Duke OCT [28]	384	Bioptigen system
Data on OCT and Fundus Images [29]	26	TOPCON’S 3D OCT-1000 system
Retina OCT Glaucoma dataset [30]	624	Cirrus SD-OCT Scanner
OLIVES Dataset [31]	96	Heidelberg Spectralis SD-OCT imaging system (Heidelberg Engineering)
Duke 2015 [32]	NA	Heidelberg Spectralis SD-OCT imaging system (Heidelberg Engineering)
2014 Srinivasan [33]	10	Heidelberg Spectralis SD-OCT imaging system (Heidelberg Engineering)
2012 Fang [34]	17	SD-OCT imaging system (Bioptigen)
OCT MS and HC data [35]	35	Heidelberg Spectralis SD-OCT imaging system (Heidelberg Engineering)
OIMHS [36]	119	Heidelberg Spectralis SD-OCT imaging system (Heidelberg Engineering)
Retouch dataset [37]	NA	OCT devices Cirrus, Heidelberg Spectralis SD-OCT imaging system (Heidelberg Engineering), and Topcon
University of Auckland Dataset [38]	NA	NA
Retinal image dataset [39]	161	Heidelberg Spectralis SD-OCT imaging system (Heidelberg Engineering)
THOCT1800 [40]	NA	NA
OCHID: An OCT Choroid Segmentation Dataset [41]	10	Heidelberg Spectralis SD-OCT imaging system (Heidelberg Engineering)
CLOUD Dataset [42]	16	OCT Cirrus 500 (Carl Zeiss Meditec)
OCTDL: Optical Coherence Tomography Dataset for Image-Based Deep Learning Methods [43]	821	Optovue Avanti RTVue XR

## Data Availability

This study did not involve the creation or analysis of new data; thus, data sharing does not apply to this article.

## Abbreviations

The following abbreviations are used in this manuscript:

OCT	Optical Coherence Tomography
AI	Artificial intelligence
DL	Deep Learning
FFA	Fundus fluorescein angiography
OCTA	Optical coherence tomography angiography
CFP	Color Fundus Photography
ICGA	Indocyanine green angiography
CSLO	Confocal scanning laser ophthalmoscopy (CSLO)
AMD	Age-related macular degeneration
DR	Diabetic retinopathy
DME	Diabetic macular edema
DE	Diabetic eye
CNV	Choroidal neovascularization
MH	Macular hole
CSR	Central serous retinopathy
MacTel	Macular telangiectasia
RP	Retinis pigmentosa
Acute CSR	Acute central serous chorioretinopathy
Chronic CSR	Chronic central serous chorioretinopathy
NA	Not applicable
MS	Multiple Sclerosis
IRF	Intraretinal fluid
SRF	Subretinal fluid
PED	Pigment epithelial detachment
RVO	Retinal vein occlusion
RAO	Retinal artery occlusion
VID	Vitreomacular interface
ERM	Epiretinal membrane

## Appendix A

The appendix contains the links for access to the datasets.

**Table A1:** Additional information for access.

Dataset Name	Link	Accessed Date
Kermany et al. [21]	https://data.mendeley.com/datasets/rscbjbr9sj/3	3 May 2024
The retinal OCT images of rare diseases [22]	https://data.mendeley.com/datasets/btv6yrdbmv/1	3 May 2024
OCTID [23]	https://borealisdata.ca/dataverse/OCTID	3 May 2024
Corneal-OCT Dataset [24]	https://sites.google.com/site/hosseinrabbanikhorasgani/available-datasets/corneal-oct?authuser=0	3 May 2024
A Composite Retinal Fundus and OCT Dataset [25]	https://data.mendeley.com/datasets/trghs22fpg/1/	3 May 2024
AROI database [26]	https://ipg.fer.hr/ipg/resources/oct_image_database	3 May 2024
Labeled Retinal OCT Dataset for Classification [27]	https://data.mendeley.com/datasets/8kt969dhx6/1	3 May 2024
Duke OCT [28]	https://people.duke.edu/~sf59/RPEDC_Ophth_2013_dataset.htm	3 May 2024
Data on OCT and Fundus Images [29]	https://data.mendeley.com/datasets/2rnnz5nz74/1	3 May 2024
Retina OCT Glaucoma dataset [30]	https://zenodo.org/records/7957454	3 May 2024
OLIVES Dataset [31]	https://zenodo.org/records/7105232	3 May 2024
Duke 2015 [32]	https://people.duke.edu/~sf59/Chiu_BOE_2014_dataset.htm	3 May 2024
2014 Srinivasan [33]	https://people.duke.edu/~sf59/Srinivasan_BOE_2014_dataset.htm	3 May 2024
2012 Fang [34]	https://people.duke.edu/~sf59/Fang_BOE_2012.htm	3 May 2024
OCT MS and HC data [35]	https://medic.rad.jhmi.edu/index.php?title=OCT_Data	3 May 2024
OIMHS [36]	https://springernature.figshare.com/articles/dataset/OIMHS_dataset/23508453	3 May 2024
Retouch dataset [37]	https://retouch.grand-challenge.org/Home/	3 May 2024
University of Auckland Dataset [38]	NA, Corresponding author	3 May 2024
Retinal image dataset [39]	NA, Corresponding author	3 May 2024
THOCT1800 [40]	https://github.com/SJD095/OCT-Segmentation	3 May 2024
OCHID: An OCT Choroid Segmentation Dataset [41]	https://imed.nimte.ac.cn/OCHID.html	3 May 2024
CLOUD dataset [42]	http://www.varpa.es/research/ophtalmology.html	3 May 2024
OCTDL: Optical Coherence Tomography Dataset for Image-Based Deep Learning Methods [43]	https://data.mendeley.com/datasets/sncdhf53xc/4	6 May 2024
